# Building the Next Generation of Humanized Hemato-Lymphoid System Mice

**DOI:** 10.3389/fimmu.2021.643852

**Published:** 2021-02-22

**Authors:** Tijana Martinov, Kelly M. McKenna, Wei Hong Tan, Emily J. Collins, Allie R. Kehret, Jonathan D. Linton, Tayla M. Olsen, Nour Shobaki, Anthony Rongvaux

**Affiliations:** ^1^Clinical Research Division, Program in Immunology, Fred Hutchinson Cancer Research Center, Seattle, WA, United States; ^2^Graduate Program in Molecular and Cellular Biology, University of Washington, Seattle, WA, United States; ^3^Medical Scientist Training Program, University of Washington, Seattle, WA, United States; ^4^Department of Immunology, University of Washington, Seattle, WA, United States

**Keywords:** humanized mice, immunity, hematopoiesis, infectious diseases, cancer, immunotherapies

## Abstract

Since the late 1980s, mice have been repopulated with human hematopoietic cells to study the fundamental biology of human hematopoiesis and immunity, as well as a broad range of human diseases *in vivo*. Multiple mouse recipient strains have been developed and protocols optimized to efficiently generate these “humanized” mice. Here, we review three guiding principles that have been applied to the development of the currently available models: (1) establishing tolerance of the mouse host for the human graft; (2) opening hematopoietic niches so that they can be occupied by human cells; and (3) providing necessary support for human hematopoiesis. We then discuss four remaining challenges: (1) human hematopoietic lineages that poorly develop in mice; (2) limited antigen-specific adaptive immunity; (3) absent tolerance of the human immune system for its mouse host; and (4) sub-functional interactions between human immune effectors and target mouse tissues. While major advances are still needed, the current models can already be used to answer specific, clinically-relevant questions and hopefully inform the development of new, life-saving therapies.

## Introduction

Biomedical research aims to provide a platform for the development and testing of new therapies that can reduce human suffering and deaths. *In vitro* studies using human cells or organoids are useful, but animal models can better elucidate the fundamental principles of complex biological processes in mammals. In particular, laboratory mice are often the model organism of choice, as their small size and short generation time enable extensive genetic engineering and invasive experimentation. Many fundamental characteristics of hematopoietic and immune systems are shared across mice and humans. However, with 91 million years of divergent evolution, differences exist and results from murine studies cannot always be directly translated into clinical applications, driving the development of experimental murine platforms that faithfully model human physiology and diseases. Specifically, mice can be transplanted with a human hemato-lymphoid system (1). Such “humanized mice” (Box 1) have been increasingly used since the late 1980s, and have contributed to major breakthroughs in several research fields, including human hematopoiesis (2), hematologic malignancies (3), and immunity to human-tropic pathogens (4, 5). Successful generation of humanized mice requires: (i) a source of human donor hematopoietic cells, (ii) an effective transplantation protocol, and (iii) an appropriate recipient mouse strain.

Box 1 **What is a humanized mouse?**

The Wikipedia definition of a humanized mouse is “a mouse carrying functioning human genes, cells, tissues, and/or organs.” This review focuses on mice transplanted with human hematopoietic cells, colloquially referred to as “humanized mice” (6, 7). When used to study immune responses, such mice are better designated as “human(ized) immune system” (HIS) mice (5, 8, 9). However, not all hematopoietic cells contribute to the immune response, and “human(ized) hemato-lymphoid system” (HHLS) mice is a more encompassing term, particularly when human CD34^+^ HSPCs are transplanted (1, 10, 11).

Many models combine multiple characteristics listed in the definition of humanized mice. In addition to human hematopoietic cell transplantation, diverse human tissues or tumors can be co-transplanted and the genome of the recipient mouse can be engineered to contain human genes.

Human peripheral blood mononuclear cells (PBMCs) can be used as a source of hematopoietic cells for transplantation, but their engraftment favors T cell maintenance, resulting in an incomplete human immune system (7, 12). In contrast, hematopoietic stem and progenitor cells (HSPCs), enriched in the CD34^+^ cell fraction, give rise to all human hematopoietic lineages upon transplantation in mice (7, 13). Human CD34^+^ HSPCs can be obtained from different sources. Fetal CD34^+^ cells, abundant in the liver, very efficiently engraft and undergo multilineage differentiation upon transplantation in mice, but ethical concerns limit access to fetal tissues (14). Other sources of CD34^+^ cells, such as cord blood, bone marrow (BM) or peripheral blood following granulocyte colony-stimulating factor (G-CSF) mobilization, are more accessible with fewer ethical concerns. However, their stemness declines with the age of the donor (15, 16), resulting in lower engraftment potential (17, 18). Of note, donor cells can be obtained from patients affected by diverse hematopoietic diseases, thereby providing small animal models of human diseases including genetic immune disorders or hematological malignancies (3, 19).

**Figure 1:**
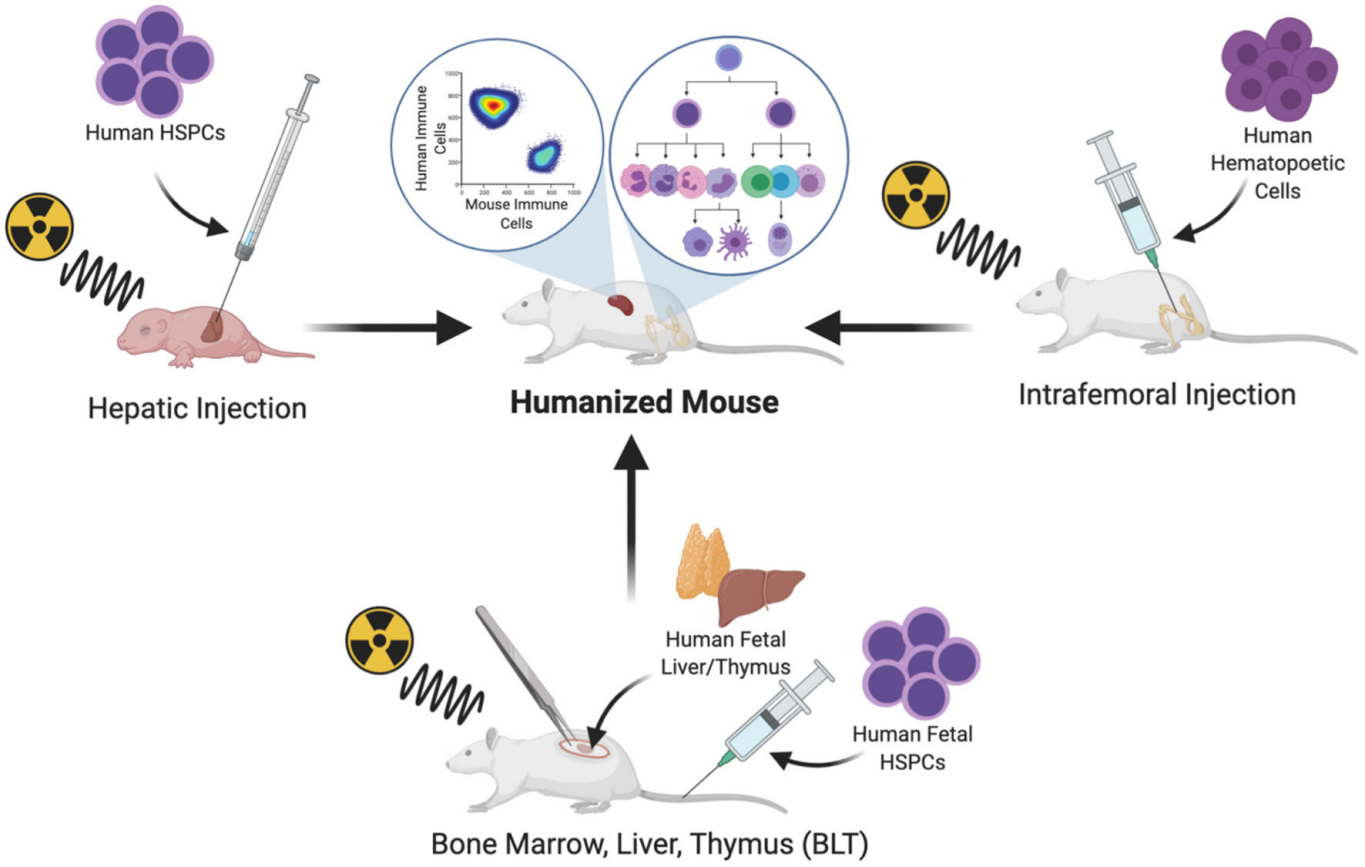
Protocols commonly used for the generation of humanized mice.

Human hematopoietic cells can be transplanted by systemic intravenous delivery or by orthotopic injection into a site of primary hematopoiesis. In adult mice, hematopoietic cells can be implanted in the BM niche by intrafemoral injection (20). Intrahepatic injection has also become a common route of CD34^+^ cell implantation in newborn mice; the liver is a site of primary hematopoiesis during embryonic life and continues to be for several days after birth, until the hematopoietic niche is established in the BM and the mouse host naturally supports the expansion and multilineage differentiation of the hematopoietic system (21) (Figure 1).

**Figure 2:**
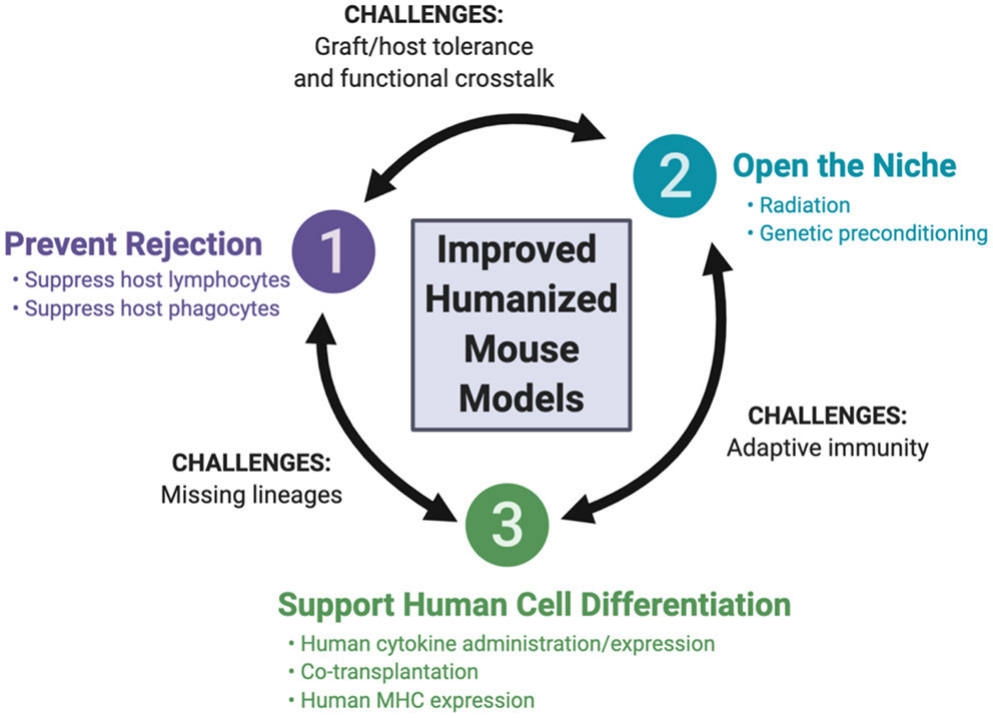
Fundamental principles of mouse humanization and remaining challenges.

The array of recipient mice for hematopoietic humanization has expanded over the past few decades, with advances in mouse genome engineering. This review focuses on these developments, highlighting the genetic engineering of the host, as well as the co-transplantation of human tissues to support human hematopoiesis. We first discuss three guiding principles that have been employed for the development of the currently available recipient mice (Figure 2): (i) preventing rejection of the human graft by the mouse immune system, (ii) opening the niche to make it accessible to human hematopoietic cells, and (iii) supporting human hematopoiesis in the mouse. We then consider how these principles are being applied to the development of newer mouse strains, aiming to resolve four remaining major challenges: (i) the development and function of missing human hematopoietic lineages, (ii) efficient and durable antigen-specific adaptive immunity, (iii) tolerance of the engrafted human immune system for the mouse host and (iv) functional cross-reactivity between the human graft and target tissues.

## Principle #1: Preventing Rejection of the Human Graft

The field of humanized mice was launched in the late 1980s, a few years after the discovery of mice with severe combined immunodeficiency (SCID). *Prkdc*^*scid*^ (protein kinase, DNA activated, catalytic polypeptide; severe combined immunodeficiency) is a spontaneous mutation identified in a colony of C.B-17 mice (22). The functional inactivation of the PRKDC enzyme in SCID mice leads to defective DNA repair and repair-dependent somatic V(D)J recombination of B and T cell receptor-encoding genes (23). As a result, lymphocyte development is arrested at an early stage and mature B and T lymphocytes are absent in SCID mice. Taking advantage of the severe immunodeficiency of these animals, several groups successfully transplanted human PBMCs (12), human BM cells (24), human fetal tissues (25) or human HSPCs (26) in SCID (12, 25, 26) or equivalent recipient mice (24). Coinciding with the early years of the HIV-1/AIDS epidemic, these pioneering models provided a much-needed tool for *in vivo* studies of HIV-1 infection (27–29). Recipient mouse strains have since undergone numerous iterative improvements, and this historical perspective has been comprehensively reviewed previously [e.g., (6)]. The most notable modifications include further preventing rejection of the human graft, through the elimination of endogenous natural killer (NK) cells and the induction of phagocytic tolerance, as discussed below.

NK cells are lymphoid cells that eliminate cells lacking major histocompatibility complex class I (MHC-I) molecules (30). Engrafted human cells express human MHC-I molecules that are not recognized by mouse NK cells. Therefore, depletion of mouse NK cells is essential, to prevent them from recognizing and eliminating the graft as “missing self.” The interleukin-2 receptor γ chain (IL-2Rγ, encoded by *Il2rg*) is shared by multiple cytokines of the IL-2 family, including IL-15 that is essential for NK cell development (31). Consequently, *Il2rg* deficiency eliminates host NK cells and improves human hematopoietic cell engraftment in immunodeficient recipient mice (21, 32–35).

Transplanted human cells are also rejected through phagocytosis by mouse cells, such as monocytes and macrophages. Because phagocytes are essential for normal development and physiology, they cannot be easily depleted genetically without affecting mouse health and survival (36, 37). An alternative strategy is to alter their functional properties, by inducing phagocytic tolerance through the signal regulatory protein alpha (SIRPα) and CD47 axis (38). The polymorphic *Sirpa* gene in the non-obese diabetic (NOD) mouse strain encodes a variant of the SIRPα receptor that cross-reacts with the human CD47 ligand. As a result, human cells transplanted in NOD mice can engage the CD47/SIRPα “don't eat me” signal and are protected from phagocytosis by mouse macrophages (39). Consequently, backcrossing the *scid* mutation, and later the *Il2rg* deficiency, onto the NOD background significantly increased the efficiency of human cell transplantation (33–35, 40, 41). The resulting strains, NOD SCID *Il2rg*^−/−^ (NOG and NSG), became very popular as they combine T, B and NK cell deficiencies with SIRPα-mediated phagocytic tolerance (33–35), but multiple other strains are functionally equivalent. These different strains abrogate V(D)J recombination [*Prkdc*^*scid*^ mutation (12, 25), or deletion of recombination activating gene (RAG)-1 or RAG-2 (21, 32, 42)] and IL-2Rγ [*Il2rg* gene deletion or truncation (21, 32–35)]. SIRPα/CD47-dependent cross-species tolerance can be achieved by expressing the mouse *Sirpa*^*NOD*^ variant (43, 44) or human *SIRPA* (45–47), or by employing a *Cd47* deficiency that produces tolerance by an unknown mechanism (48, 49). These strains are on diverse genetic backgrounds (NOD, BALB/c, C57BL/6), and are known by distinct acronyms, listed in Table 1. Upon transplantation of human CD34^+^ HSPCs, all of these strains support the differentiation of high levels of human CD45^+^ cells, reaching about 80% engraftment in the BM and 50% in the periphery. However, immune cell differentiation is disproportionately skewed toward the B and T lymphoid lineages (33, 35, 45). Human myelo-monocytic and NK cells are present only at low frequencies (50, 51), and human circulating red blood cells and platelets are barely detectable (52, 53). Investigators should carefully select the background strain they use, based on their research question, as the specific strain can impact the outcome of experiments. For example, SCID mice are highly susceptible to DNA damage; therefore RAG-deficient mice are the preferred recipient strain when testing chemo- and radiotherapies (42, 54). For studies involving complement-dependent cytotoxicity, the C57BL/6 or BALB/c backgrounds should be preferred, since NOD mice lack hemolytic complement C5 (41, 44).

**Table 1:** List of immunodeficient mice used as recipients for transplantation of human hemato-lymphoid system.

**Acronym**	**Genetic** **background**	**T and B cell** **deficiency**	**NK cell** **deficiency**	**Phagocytic** **tolerance**	**References**
SCID	C.B-17	*Prkdc^*scid*^*	-	-	(12, 25, 26)
NOD-SCID	NOD	*Prkdc^*scid*^*	-	*Sirpa^*NOD*^*	(40, 41)
BRG	Balb/c	*Rag2^−/−^*	*Il2rg^−/−^*	-	(21, 32)
NOG	NOD	*Prkdc^*scid*^*	*Il2rg^−/−^* (truncation)	*Sirpa^*NOD*^*	(35)
NSG	NOD	*Prkdc^*scid*^*	*Il2rg^−/−^*	*Sirpa^*NOD*^*	(33, 34)
NRG	NOD	*Rag1^−/−^*	*Il2rg^−/−^*	*Sirpa^*NOD*^*	(42)
BRGS^NOD^	Balb/c	*Rag2^−/−^*	*Il2rg^−/−^*	*Sirpa^*NOD*^*	(43)
S^tg^RG	Balb/c x 129	*Rag2^−/−^*	*Il2rg^−/−^*	Human *SIRPA* (BAC tg)	(45)
S^KI^RG	Balb/c x 129	*Rag2^−/−^*	*Il2rg^−/−^*	Human *SIRPA* (KI extracellular domain)	(46)
B6RGS^NOD^	C57BL/6	*Rag2^−/−^*	*Il2rg^−/−^*	*Sirpa^*NOD*^*	(44)
B6RGS^Human^	C57BL/6	*Rag2^−/−^*	*Il2rg^−/−^*	Human *SIRPA* (KI full-length)	(47)
B6RG-CD47	C57BL/6	*Rag2^−/−^*	*Il2rg^−/−^*	*Cd47^−/−^*	(48)

*SCID, NOD-SCID and BRG are historical models, that provided the basis for most currently used recipient mice. All other mice listed in this table have conceptually similar properties, with a few exceptions, as discussed in the text*.

## Principle #2: Opening the Niche

Hematopoiesis is a complex and tightly regulated process during which hematopoietic progenitors undergo expansion and multilineage differentiation (2, 13). This process occurs primarily in the BM that uniquely provides supporting factors, such as cytokines at local physiological concentrations, and also provides a distinct microenvironment for developing cells (55). Accessibility of the transplanted human CD34^+^ HSPCs to this niche is required for efficient engraftment in the mouse host. Reducing cellularity in the mouse BM creates the needed physical space, described as “opening the niche.” Traditional protocols rely on irradiation as a preconditioning regimen (56–60), typically achieved using sub-lethal X-ray or ^137^Cs irradiation to kill most hematopoietic cells while limiting toxicity. When no irradiator is available, alternative pre-conditioning protocols can be used, such as the myeloablative drug busulfan (61–63).

Recently, the requirement for radiation preconditioning has been alleviated with recipient mice engineered to have a less populated BM niche, thereby achieving a form of “genetic preconditioning.” An example is provided by the *Kit*^*W*41^ mutation, in an NSG-derived mouse strain known as NSGW41 (64) (Table 2). The receptor tyrosine kinase Kit (also known as c-Kit or CD117, encoded by the *Kit* gene) is the receptor for the cytokine stem cell factor (or SCF, also known as steel factor or Kit ligand). SCF increases HSPC retention in the BM niche by increasing their adhesion to neighboring stromal cells and proteins in the extracellular matrix (75). The *Kit*^*W*41^ allelic variant encodes a protein with partially impaired kinase activity (76–78), producing functionally defective hematopoietic stem cells in *Kit*^*W*41/*W*41^ mutant mice. In addition, because SCF is conserved between species [over 82% amino acid identity between mouse and human (1)], mouse SCF cross-reacts on the corresponding human Kit receptor. Consequently, when transplanted into NSGW41 mice, human HSPCs find a partially open BM niche and effectively compete for SCF against the mouse HSPCs that express the impaired Kit receptor. The impact of the *Kit*^*W*41/*W*41^ mutation on mouse humanization is threefold: human CD34^+^ HSPCs efficiently engraft without radiation preconditioning; even without radiation, engraftment levels in NSGW41 are higher than in irradiated NSG recipients; and better maintenance of functional human HSPCs favors their multilineage differentiation, including into the erythro-megakaryocytic lineage (64, 79).

**Table 2:** List of genetically engineered mice, in the order discussed in this review.

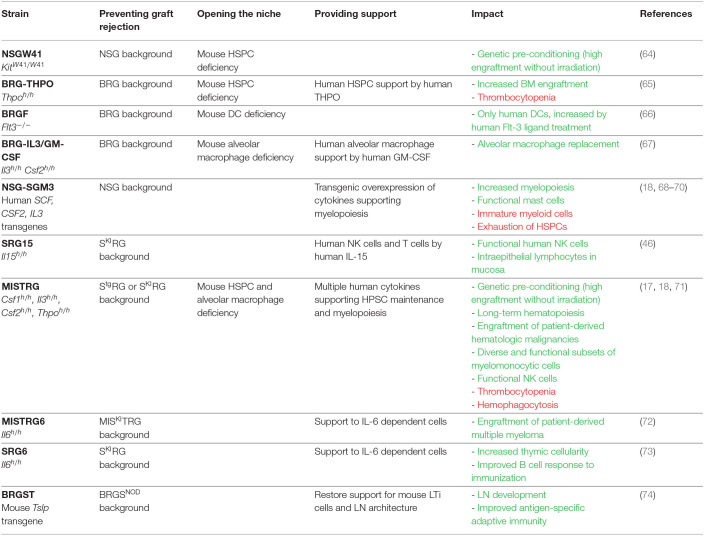

*Positive impacts are indicated in green, unintended detrimental effects in red. A more detailed version is provided as Supplementary Table 1, including additional details of improvements and limitations of each model with regards to hematopoiesis, innate and adaptive immunity*.

Other genes can be inactivated to open the BM niche. *Thpo* encodes the cytokine, thrombopoietin, which is essential for the maintenance of quiescent and self-renewing HSPCs (80–83). Mouse *Thpo* gene inactivation reduces frequencies of mouse HSPCs, thereby opening the niche for transplanted human HSPCs (65). The concept of genetic preconditioning also applies to non-HSPC cell types, and to niches other than the BM. Fms-like tyrosine kinase 3 (Flt-3) ligand is essential to the differentiation of dendritic cells (DCs) (84–87), while granulocyte-macrophage colony stimulating factor (GM-CSF) is required for the maturation of lung alveolar macrophages (AM) (88–90). Genetic inactivation of *Flt3* (encoding the receptor for Flt-3 ligand) or of *Csf2* (encoding GM-CSF) eliminates mouse DCs or AMs, respectively, thereby opening the niche for the development of the corresponding human cell lineages (66, 67). In these three cases (*Thpo, Ftl3*, or *Csf2* gene deficiencies), the elimination of mouse cell populations was supplemented with provision of the corresponding human cytokines (65–67), as discussed next.

## Principle #3: Supporting the Development of Engrafted Human Cells

The spatial microenvironment of the BM niche includes diverse cell types, including endothelial cells and mesenchymal stromal/stem cells (MSCs) that are known to release cytokines, signaling mediators and growth factors, such as SCF, IL-3, IL-6, THPO, and GM-CSF (91–93). These molecules are important for the maintenance of human HSPCs and their differentiation into all hematopoietic and immune cell lineages (1, 2). Some of the cytokines supporting hematopoiesis are poorly conserved (e.g., 29% amino acid identity between human and mouse IL-3) and do not cross-react from mouse to human, while others are highly conserved and largely cross-reactive (e.g., SCF) (1). However, even when amino acid identity is high, cross-reactivity of cytokines is not always complete in local microenvironments at physiological concentrations. To account for this incomplete cross-reactivity, various protocols were developed to promote the differentiation of a more complete human immune system upon human CD34^+^ cell transplantation in mice.

### Exogenous Cytokine Administration

The simplest method of supplying graft-supporting factors is the repeated injection of recombinant human cytokines. This approach was used in an early-generation SCID model and the injection of cytokines, including human SCF, IL-3, and GM-CSF, supported enhanced engraftment levels of human BM cells as well as their myeloid differentiation (26). Because human NK cell differentiation is limited in mice, humanized BRG mice were treated with recombinant human IL-15 coupled to IL-15Rα, which is an essential growth factor for NK cell development and homeostasis. This treatment resulted in a significant increase in the differentiation and homeostasis of human NK cells in mice transplanted with human CD34^+^ cells (94).

A second cytokine delivery approach relies on hydrodynamic injection of a human cytokine-encoding DNA plasmid, resulting in the *in vivo* “transfection” of hepatocytes. In turn, hepatocytes produce high levels of the encoded cytokine and release it into the circulation for up to 5 days (95, 96). This method was used to demonstrate that human cytokines, such as GM-CSF and M-CSF, support myeloid differentiation of human CD34^+^ cells in NSG mice, while IL-15 and Flt-3 ligand supported NK cell differentiation (96, 97).

The administration of human cytokines can be combined with genetic opening of the niche. In BRGF mice, which lack the *Flt3* gene, injection of human recombinant Flt-3 ligand boosts the development of human dendritic cells, in the absence of mouse dendritic cells (66).

Recombinant cytokine injection and hydrodynamic plasmid delivery are easy to implement, irrespective of the recipient mouse and protocol of human cell transplantation used. However, they both result in transient, systemic and generally supra-physiological cytokine expression.

### Genetic Engineering of Human Cytokine Expression

To circumvent the requirement for repeated cytokine administrations, the genome of the recipient mouse can be engineered to express human cytokines (Table 2). The initial method relied on transgenic overexpression of a human cytokine-encoding cDNA, under the control of a strong promoter. Such transgenic mice are still in use, based on NSG or similar genetic backgrounds. However, results obtained with these mice need to be interpreted cautiously as the systemic overexpression of human cytokines frequently results in non-physiological hematopoiesis. For example, the transgenic pCMV promoter-driven overexpression of human SCF, GM-CSF, and IL-3 results in the mobilization of CD34^+^ HSPCs in the NSG-SGM3 mouse and the loss of their functional properties (68). As a consequence, although high-level human hematopoietic engraftment is achieved in NSG-SGM3 mice, hematopoietic progenitors lose their stemness and long-term hematopoiesis is deficient, as demonstrated by their inability to serially engraft (18, 69). Nevertheless, transgenic overexpression of cytokines can support the development of specific cell lineages and provide useful models to study those cells. In NSG-SGM3, the overexpression of human SCF results in the development of abundant and functional human mast cells, which are effective in models of passive cutaneous and systemic anaphylaxis (98). Similarly, human IL-15 overexpression in NOG mice supports the maintenance and function of human NK cells isolated from human peripheral blood (99, 100). Such models can provide useful experimental systems to evaluate the effect of candidate drugs on specific human immune cell populations.

**Figure 3:**
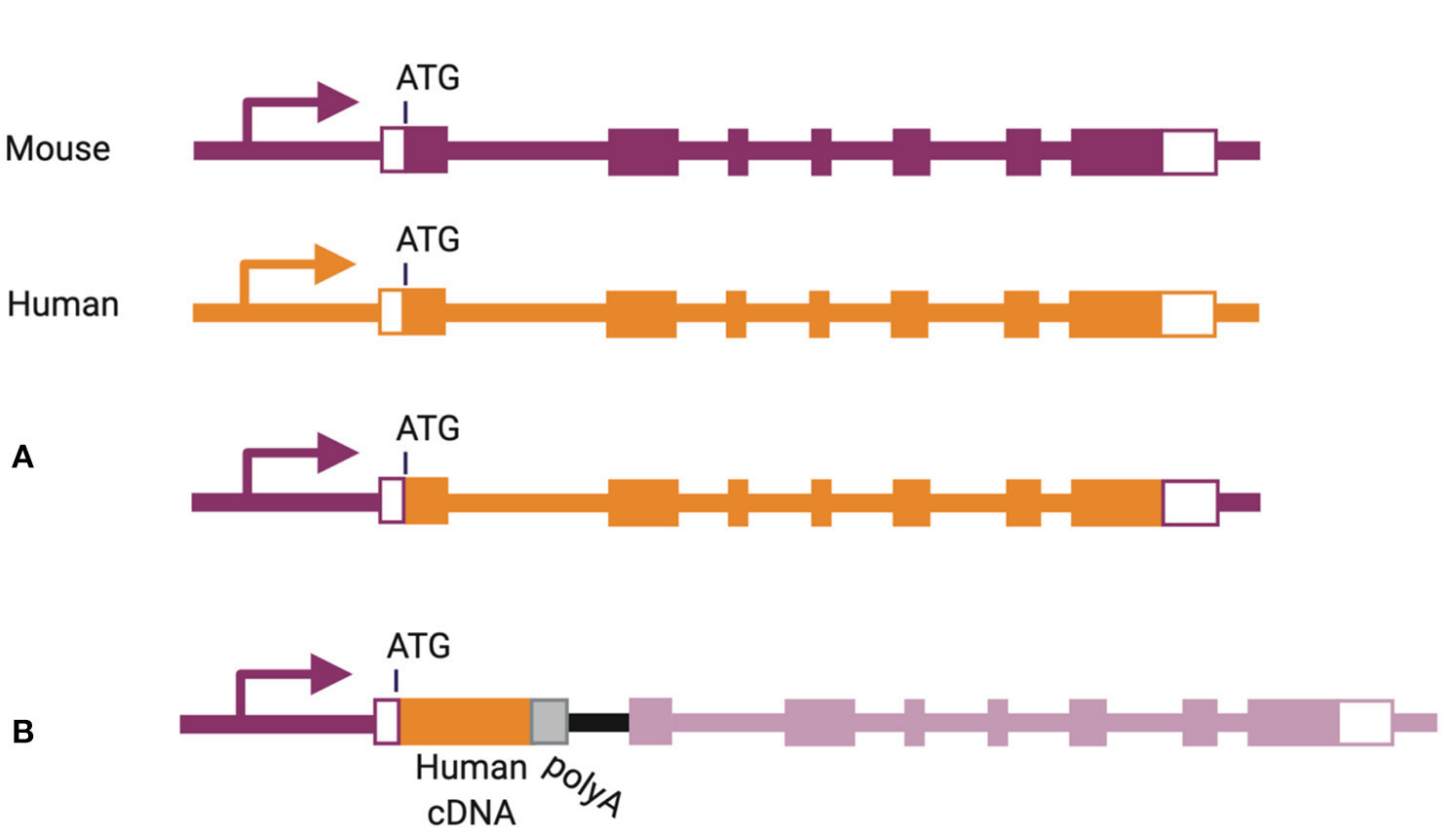
Methods of knock-in gene humanization by **(A)** gene replacement or **(B)** cDNA insertion.

More physiological expression of human cytokines can be achieved by bacterial artificial chromosome (referred to as “BAC”) transgenesis, inserting an entire human gene, including its regulatory elements, into the mouse genome. This method has been used to express human IL-6 and human IL-7 in NSG mice (101, 102). In another strategy, the mouse gene can be eliminated and corresponding human gene inserted in its place, including introns and exons from the start to the stop codon (Figure 3A) (103). This knock-in strategy has been used for a number of cytokine-encoding genes, including *Csf1, Csf2, Il3, Il6, Il15*, and *Thpo* (46, 65, 67, 72, 104). A slightly different approach was used for replacement of the *Il7, Il15*, and *Tnfsf13b* genes, through insertion of a human cDNA in frame with the start codon of the corresponding mouse gene, followed by a poly-adenylation signal (Figure 3B) (101, 105).

Because of its role in supporting NK cell development, human IL-15 is highly featured in the humanized mouse literature, and it has been delivered to recipient mice by each of the methods described above (46, 94, 96, 99, 101). Since these approaches have been reported by different groups, their direct comparison and the evaluation of respective merits and limitations are difficult. However, the SRG-15 recipient mouse stands out as a definitive solution to the limited development of human NK cells in humanized mice, owing to its simplicity of use (no cytokine administration is needed), the physiological expression of the cytokine by knock-in gene replacement, the comprehensive phenotypic comparison to human peripheral blood cells and rigorous functional *in vivo* characterization (46).

In addition to expressing a human cytokine, knock-in gene replacement abrogates expression of the corresponding mouse cytokine (Figure 3). If the human cytokine is not fully cross-reactive, this can result in the absence of the cytokine-dependent mouse cell population and niche opening (103). This is best illustrated in the case of GM-CSF (encoded by *Csf2/CSF2*), where only 56% of amino acids are shared between human and mouse (1, 67). In mice with homozygous humanization of the *Csf2* locus, the absence of mouse GM-CSF induces alveolar macrophage deficiency in the lung and a resulting pathology described as pulmonary alveolar proteinosis (67), recapitulating the phenotype of *Csf2* knockout mice (88–90). Upon human CD34^+^ cell transplantation, human GM-CSF supports the development of human alveolar macrophages and partial rescue of the proteinosis pathology (67).

Importantly, because hematopoiesis is a stepwise process in which stem cells gradually differentiate toward more committed progenitors and multiple lineages of mature cells (1, 2), humanization of several cytokines is required to support the maintenance and multilineage differentiation of human HSPCs. This is exemplified in the MISTRG mouse in which M-CSF, IL-3, GM-CSF and THPO are humanized by knock-in replacement, on the SRG genetic background (17, 71). Niche opening and four human cytokines synergize to support the engraftment of human CD34^+^ cells without radiation preconditioning (17, 18). The long-term maintenance of functional HSPCs is demonstrated by their serial transplantation for up to four generations of MISTRG recipients (18). These mice also support multilineage differentiation of human B, T and dendritic cells (similarly to NSG mice) as well as different subsets of functional myelo-monocytic cells in lymphoid and non-lymphoid tissues (17, 18). The myelo-monocytic cells themselves express human IL-15/IL-15Rα, which in turn supports the development and function of human NK cells (17). Overall, MISTRG mice emphasize the benefits of combining multiple strategies for the provision of human cytokines in the development of improved recipient mice by: niche opening by elimination of the corresponding mouse cytokines; physiological expression of each cytokine; synergy between multiple cytokines; and, indirectly, cross-support between different human immune cell lineages.

### Co-transplantation of Supporting Human Cells or Tissues

Although providing specific cytokines can be effective, it would be a long and arduous journey to humanize the entire spectrum of hematopoiesis-supporting cytokines (and possibly other required factors). Therefore, co-transplantation of supporting human cells or tissues, naturally found in the BM niche or in other primary lymphoid organs, can be used to provide all required factors and more fully recapitulate human hematopoietic development and homeostasis.

A well-established protocol of such co-transplantation is the BM, liver, thymus (BLT) humanized mouse model (106, 107). BLT mice are generated by co-transplanting human fetal liver and thymus under the murine renal capsule in preconditioned adult immunodeficient mice, before injecting syngeneic CD34^+^ HSPCs intravenously. The liver and thymus implants form a liver-thymus organoid that contains stromal microenvironments and provides cytokines at local physiological concentrations, requisite for the differentiation of functional B, T, and NK cells, dendritic cells and monocytes/macrophages, as well as long-term maintenance of human hematopoiesis and lymphopoiesis (106–108). Because human T cells develop in the context of thymic epithelial cells, the human thymus organoid contains significantly higher absolute numbers of thymocytes, compared to mouse thymus (109) and a repertoire of immunocompetent human leukocyte antigen (HLA) class I- and class II-restricted T lymphocytes is selected (106, 107, 110). The immune cell distribution in BLT humanized mice is well-described for both primary and secondary lymphoid organs, including BM, human and mouse thymus, intestines (lamina propria), mesenteric lymph nodes, vaginal tissues, liver and lungs (106, 107, 109, 111–113). Although the BLT protocol was first described using the NOD-SCID background (106, 107), this technique can be applied to any recipient mouse, and has been used successfully in NSG (109, 113), NSG-SGM3 (98), and B6RG-CD47 (48) mice. Thus, investigators can use the BLT protocol to enhance human T cell development in a mouse strain with immune characteristics that are appropriate for their study.

Intravenous or intrafemoral co-transplantation of human BM-derived MSCs along with CD34^+^ cord blood cells is another strategy to improve engraftment of CD34^+^ and CD45^+^ cells in immunodeficient mice (114–117). Overall, co-transplantation of the BM-derived MSCs into humanized mice can improve human HSPC maintenance and expansion, and improve reconstitution of the human hemato-lymphoid system in humanized mice. Furthermore, recent studies have used genetically engineered MSCs to deliver additional human factors to support human hematopoiesis, or to facilitate the expansion and maintenance of HSPCs to allow serial transplantation and generation of larger quantities of humanized mice (118, 119).

Recent studies have used human bone organoid (ossicles) as a method to add human BM microenvironment for engrafted HSPCs to occupy (120, 121). Ossicles are often generated by seeding 3D polymer scaffolds with human BM-derived MSCs. These are then implanted subcutaneously into NSG mice and become colonized with subsequently injected human HSPCs, enabling their expansion and differentiation (122–128). Humanized ossicles contain increased human immature and mature hematopoietic cells as compared to the bones of host mice implanted with only human CD34^+^ cells, indicating homing of HSPCs (122, 128). Additionally, self-renewing HSPCs from humanized ossicles can reconstitute hematopoiesis in secondary recipient mice, demonstrating maintenance of their functional properties (129). Most importantly, high engraftment levels of hCD45^+^ cells were measured in the blood, spleen and mouse bone (130, 131). High numbers of human erythroid lineage cells and robust differentiation of mature myeloid cells were also detected (132).

With the development of MISTRG and MISTRG6 mouse strains that express essential human cytokines, and protocols for co-transplantation of human fetal bone chips or ossicles, major progress has been made in transplanting patient-derived hematologic malignancies into humanized BM niches. Samples from patients with myelodysplastic syndromes, myeloproliferative neoplasms, low risk acute myeloid leukemia, diffuse large B cell lymphoma or multiple myeloma have been successfully engrafted, using diverse recipient mice and protocols (72, 123, 125, 133–136).

## Future Challenge #1: the Missing Lineages

Provision of specific human cytokines significantly improved the development, homeostasis and function of human NK cells and myelo-monocytic cells in humanized mice. But several lineages remain defective. For example, human neutrophils are generally present in the BM of humanized mice, but their frequency is negligible in the periphery (50). Cytokine overexpression in NSG-SGM3 mice increases the frequency of granulocytic CD33^+^ cells in the BM and the periphery (18, 69, 70), but these cells display the morphology and cell surface phenotype of immature cells (18). Human cytokine knock-in MISTRG mice also do not have improved mature neutrophil numbers in the periphery (17, 18). Therefore, the differentiation, egress, maturation and/or survival of human neutrophils likely requires additional factors.

Human red blood cells (RBCs) and platelets are probably the most challenging hematopoietic cells to develop in mice. They illustrate that additional strategies, beyond the provision of human cytokines, will likely be needed to support the complete spectrum of human hematopoietic lineages in mice. In the BM of humanized NSG mice, human erythroid (CD235a^+^) and megakaryocytic (CD41^+^CD61^+^) progenitors are extremely rare. Their frequency is increased by at least an order of magnitude in NSGW41 and MISTRG recipient mice but surprisingly, overexpression of human erythropoietin (EPO) did not further improve human erythropoiesis (79, 133). The increase in genetically preconditioned mice is likely due to the better competition of human progenitors against mouse progenitors in the open hematopoietic niche, and/or support from knocked-in human factors. However, erythropoiesis is arrested at an immature (CD71^+^ CD235a^+^) stage. As a result, few mature CD71^−^CD235a^+^ human reticulocytes are detectable in the BM and human RBCs rarely exceed 1% in peripheral blood (79).

Several lines of evidence demonstrate that this deficiency is due to a developmental defect as well as impaired survival. Indeed, human RBCs are highly susceptible to destruction in mice. Expression of human SIRPα in BRG mice, or depletion of macrophages by clodronate treatment in NOD SCID mice, extends the lifespan of adoptively transferred human RBCs (45). However, in both cases, the half-life of human RBCs does not exceed ~16 h, which is much shorter than the 10–20-days half-life of mouse RBCs in similar transfer experiments (137). Accordingly, clodronate treatment results in significant but transient and incomplete increase in human RBCs and platelets in mice humanized by CD34^+^ cell transplantation (52, 53, 79). In those conditions, injection of the human cytokines, IL-3 and EPO, promotes an increase in peripheral RBC counts (52). But, because clodronate targets both mouse and human cells, the treatment results in a humanized mouse entirely lacking human phagocytic cells, which limits the applicability of the model for studies of human immunity.

Therefore, the entire panel of mechanisms limiting the half-life of human RBCs and platelets in mice will need to be identified and resolved. In addition, the adequate combination of human cytokines will have to be provided, to enable the mice to live with primarily human platelets and RBCs. Such a model would be highly useful for studying diseases caused by pathogens with exclusive tropism for human RBCs [e.g., malaria caused by *Plasmodium falciparum* (138)] or diseases in which platelets contribute to pathogenesis [e.g., dengue fever, autoimmune thrombocytopenia (139, 140)]. In the meantime, current models (such as MISTRG) have already demonstrated their utility for modeling the early BM stages of human erythropoiesis and thrombopoiesis, as well as for studying drug responses in pathologies, including myelodysplastic syndromes and myeloproliferative neoplasms (133, 134).

## Future Challenge #2: Adaptive Immunity

As a result of advances discussed above, we now have humanized mice in which both cellular and humoral adaptive immune responses can be elicited. However, these responses are largely modest in magnitude, quality and duration, as outlined below.

### Antigen-Specific Adaptive Immunity

Humanized mice generated by transplantation of CD34^+^ cells, or following the BLT protocol, can mount antigen-specific adaptive immune responses (21, 107, 141, 142). Evidence of protective immunity, capable of controlling pathogen replication and resulting disease, was provided in the context of Epstein-Barr virus (EBV), dengue virus (DENV), and human immunodeficiency virus-1 (HIV-1) infection. Indeed, in CD34^+^-humanized NSG mice, antibody-mediated depletion of T cells led to the development of EBV-associated tumors, suggesting that T cell-mediated immunity can control EBV infection in this setting (143). Robust CD4^+^ and CD8^+^ T cell responses were also detected after DENV and HIV-1 infection. Notably, in HIV-1-infected BLT mice, CD8^+^ T cell responses were HLA-restricted and directed against epitopes previously described as immunogenic in humans. Furthermore, CD8^+^ T cell-mediated viral recognition led to viral epitope evolution, closely resembling clinical observations (142).

Despite having detectable anti-viral T cells, CD34^+^-humanized and BLT mice generally exhibit weak humoral responses after viral infection (144, 145). While neutralizing antibodies were detected in a subset of DENV-infected mice, in both CD34^+^-only and BLT humanized mice, the anti-EBV or anti-HIV-1 antibody responses were either weak, equivocal or delayed (111, 141). This was also the case in humanized MISTRG mice, where enhanced innate immune cell engraftment led to improved T cell function after *Listeria monocytogenes* infection, but humoral immunity remained weak (17). Across these models, immunoglobulin class-switching and somatic hypermutation are rarely achieved, likely because most B cells fail to reach a fully mature phenotype in the periphery, T cell repertoire is selected on mouse and human MHC and is suboptimal, and recipient mice have disorganized lymph nodes and little capacity for germinal center formation (146).

While human B cells are detectable in high frequencies in humanized mice, most exhibit an immature phenotype, and remain blocked at the transitional stage of B cell development (1, 146). Since immature B cells have a reduced capacity to respond to antigen (147), several groups sought to improve B cell maturation and by extension, their function by knocking-in human cytokines that are known to support B cell development. Surprisingly, human B cell activation factor (BAFF, encoded by *TNFSF13B*) knock-in resulted in reduced numbers of mature naïve B cells, and reduced antibody production after immunization (105). Human IL-7 knock-in had no effect on B cell numbers and any effect on B cell function remains to be determined (101). In contrast, human IL-6 positively impacted B cell differentiation into plasmablasts and memory cells after immunization with model antigen, and promoted somatic hypermutation and class switching, albeit to a lower level than that observed in humans (73). IL-6 knock-in mice also had improved T cell development; therefore, it is possible that enhanced B cell responses were in part due to increased T cell help.

### Thymic Lymphopoiesis and Repertoire Selection

Thymic lymphopoiesis is suboptimal in mice humanized by transplantation of CD34^+^ HSPCs, in part due to a lack of thymopoiesis-supporting human cytokines. As a result, thymic cellularity is extremely low (only a few million cells) and CD4^+^ CD8^+^ double positive cells, which represent the vast majority of thymocytes in a healthy thymus, are frequently underrepresented. Human IL-6 knock-in produces increased thymic cellularity (73), but additional cytokines are likely required to restore the normal size of a mouse thymus, populated by human thymocytes. While a human IL-7 knock-in recipient mouse has been developed, a thorough characterization of thymic cellularity upon HSPC engraftment has not been reported to date (101).

Human T cell repertoire selection in the mouse thymus involves interactions with both mouse MHC and human HLA-expressing cells-expressing cells (1, 10, 21). Specifically, developing human T cells are positively selected by mouse epithelial cells and negatively selected by both mouse epithelial and mouse and human hematopoietic cells (148). The resulting T cell receptor (TCR) repertoire is weakly reactive to autologous human leukocyte antigen (HLA) class I and II, and tolerant to mouse MHC. Indeed, in *in vitro* re-stimulation assays, T cells from humanized mice were shown to proliferate better in response to allogeneic human DCs compared to autologous human DCs or mouse DCs (21). To improve the repertoire and function of human T cells, several recipient strains have been engineered to express HLA class I and class II (143, 144, 149–154). HLA-restricted T cell responses have been reported in these mice (143, 149). Additional characterization and comparison of these mice are required to rigorously evaluate how transgenic HLA expression in recipient HSPC-humanized mice impacts human T cell selection and function.

In BLT mice, T cells develop in the human thymus organoid and are positively and negatively selected on human autologous HLA molecules (106, 107, 155). Consequently, BLT mice are generally accepted as having a more diverse T cell repertoire, capable of mounting more robust adaptive immune responses, as discussed above. However, because the induction of tolerance for mouse MHC and mouse tissue-restricted antigens is incomplete, BLT mice are prone to the development of xenogeneic graft-vs.-host disease (xGvHD) (156, 157), as discussed below.

Few direct comparisons between BLT and HSPC-engrafted mice have been reported to date (144, 145). In both models, it is apparent that thymic lymphopoiesis and the selection of a diverse and tolerant T cell repertoire is a complex issue, and additional work is needed.

### Secondary Lymphoid Organs

Secondary lymphoid organs including lymph nodes (LNs), spleen, Peyer's patches, and mucosa-associated lymphoid tissues normally provide niche microenvironments where T and B cells interact with hematopoietic antigen-presenting cells and stromal follicular dendritic cells (FDCs) to initiate the adaptive immune response (158, 159). LN formation requires IL-7- and IL-2Rγ-dependent lymphoid tissue inducer (LTi) cells (160). However, because most immunodeficient recipient mice lack *Il2rg* to promote tolerance to the human graft through NK cell depletion, they also lack LTi cells and defined LN structures. This represents a major obstacle in recapitulating the human adaptive immune response in mice. While engraftment with human CD34^+^ cells can partially rescue the LN anlagen, T and B cell zones remain poorly organized compared to those in normal human or normal mouse LNs. Furthermore, the germinal center formation is impaired, in part because it involves human B cells interacting with stromal FDCs of mouse origin (1, 159).

Given that the germinal center is the primary site of B cell-T cell collaboration, antibody affinity maturation and class switching, multiple groups sought to improve LN formation and germinal center development (158). Adoptive transfer of autologous human DCs lentivirally transduced to express GM-CSF, IFNα, and human cytomegalovirus (HCMV) viral antigen enhances LN development in humanized NRG mice, and by extension promotes T cell-B cell collaboration and the production of neutralizing anti-HCMV antibodies (161, 162). Human fetal organ co-transplantation also partially rescues secondary lymphoid organ development. BLT mice have improved lymphoid structure development in the spleen and lymph nodes compared to CD34^+^-only engrafted mice, and as a result, they also have more robust antigen-specific adaptive immunity (106, 107). However, a comparison of IL-2Rγ-sufficient NOD-SCID BLT humanized mice and IL-2Rγ-deficient NSG BLT humanized mice showed that only NOD-SCID BLT mice contain mucosal tissue-associated T cells (109). This result emphasizes the critical nature of IL-2Rγ in the development of LN and other secondary lymphoid organs that are essential for adaptive immune responses and mucosal immunity. The importance of structured secondary lymphoid organs for adaptive immunity is further demonstrated by the addition of fetal spleen to BLT mice (163). Human fetal spleen implants grow into spleen organoids with prominent follicular lymphoid structure; implanted mice have improved B cell and T cell engraftment, compared to control mice without human spleen implants, and can mount antigen-specific responses to immunization (163).

Another strategy to overcome the near-absence of LNs in *Il2rg*^−/−^ humanized mice involves transgenic overexpression of murine thymic stromal lymphopoietin (TSLP). TSLP is another cytokine of the IL-2 family, its receptor is independent of the IL-2Rγ chain and, when overexpressed under a keratin 14 promoter (specific for epithelial/mesenchymal cells), it rescues LN development in *Il7* deficient mice (164, 165). Crossing the transgene to BRGS resulted in the BRGST model, in which mouse LTi cells and LN structures are restored (74). Upon transplantation of CD34^+^ HSPCs, BRGST mice support the development of a human immune system, including sizeable LNs with compartmentalized human T and B cell zones. BRGST mice also have more mature B cells and IL-21 producing follicular helper T cells, essential to promoting adaptive immunity. Consequently, BRGST mice mounted enhanced antigen-specific humoral immune responses upon immunization with an experimental antigen (74). Overall, this novel model successfully addresses a major limitation that had hampered immune function in most humanized mouse models to date.

## Future Challenge #3: Graft-to-Host Tolerance

The transplantation of human cells into recipient mice is feasible because the recipient mice used are immunologically tolerant to the graft. As these mice support an increasingly functional human immune system, immunologic tolerance of the graft for its host can become an issue.

### Xenogeneic Graft-vs.-Host Disease

Adoptive transfer of human T cells from donor PBMCs into immunodeficient recipient mice results in xGvHD, thereby limiting the potential duration of experiments with these mice. To prevent xGvHD, two strains of immunodeficient mice lacking mouse MHC-I and MHC-II have been developed, which prevented the onset of xGvHD, while retaining the functional properties of human T cells (166, 167). But, as discussed above, PBMC transfer results in an incomplete human hematopoietic system in the mice.

When mice are humanized by transplantation of CD34^+^ HSPCs, developing human T cells undergo positive and negative selection in the mouse thymus. Consequently, they are tolerized for mouse MHC-I and MHC-II, and xGvHD is not a limitation in this model (1, 10, 21).

Finally, in BLT mice, T cells are educated in the human thymus organoid and once they reach the periphery, they are allogeneic to the mouse MHC molecules and can induce xGvHD (156, 157). Different levels of xGvHD are reported by different groups, suggesting that the disease could be affected by subtle differences in protocols, the microbiota of the mice, or the recipient strain used (48). The development of xGvHD in the BLT model is attributed to residual mature T cells present in the fetal human thymus grafts, and these passenger T cells can be removed by treating the thymic implants with 2′-deoxyguanosine, or by treating the mice with anti-human CD2 antibody post-surgery (168).

### Xenogeneic Hemophagocytosis

The efficient development of human phagocytic cells of the myelo-monocytic lineage in MISTRG creates a new challenge; i.e., the absence of phagocytic tolerance from the human graft toward the mouse host. Mouse red blood cells are particularly susceptible to destruction by human phagocytic cells, and highly engrafted MISTRG mice develop lethal anemia (17). Consequently, the transplantation protocol in MISTRG needs to be optimized so that engraftment allows mouse erythropoiesis in the BM (or extramedullary erythropoiesis) to sufficiently compensate for the loss of mouse red blood cells by phagocytosis, as long as a specific experiment requires (17, 18, 58, 133). Long-term solutions will need to be implemented, either to establish human-to-mouse phagocytic tolerance or to enable human erythropoiesis to reach healthy human RBC counts and maintain mouse homeostasis, as discussed above.

Xenogeneic GvHD and hemophagocytosis remind us that activation and tolerance are two equally important features of the immune system and are inseparable. As human immune function improves in mouse recipients, it is likely that parallel strategies will need to be developed to maintain tolerance of the human immune graft for the mouse host.

## Future Challenge #4: Interactions Between Immune Cells and Target Tissues or Tumors

Immune responses require functional interactions between immune cells and their target, either through direct cell-cell contact or via soluble factors. In humanized mice generated by transplantation of human HSPCs, this requirement is fulfilled when the target of the immune response is also a human hematopoietic cell. Accordingly, T and B cell-mediated immunity have been demonstrated in the context of infection by pathogens with tropism for human hematopoietic cells, such as EBV, HIV-1, or dengue virus (141, 143, 169). But, for pathogens that infect non-hematopoietic tissues, or for inflammatory mediators that induce systemic responses, the cross-reactivity between human immune effector mechanisms and mouse target tissues may be incomplete. Humanizing cytokine receptors or other factors, such as adhesion molecules, could improve the responsiveness of mouse tissues to human immune cells and soluble mediators.

Co-transplantation of the human target tissue along with the human hematopoietic system has been performed in the context of cancer and infectious diseases. Implantation of human tumors, either from an established cell line or from a “patient-derived xenograft” (PDX), in mice already repopulated with a human immune system, provides useful models for immuno-oncology and immunotherapy studies (170–173). However, developing such “immuno-PDX” models can be challenging as each patient-derived tumor or cell line has different growth characteristics in mice, and matching patient's HLA to the HLA of the HSPC donor is not always feasible. Additionally, many components of the tumor microenvironment (e.g., vasculature) remain of mouse origin. Consequently, antitumoral immunity, or the response to immunotherapies, can be highly variable from experiment to experiment (171). In the case of hematologic malignancies, new strains of recipient mice (i.e., MISTRG and MISTRG6) extend the range of transplantable diseases (72, 133–136) and provide new opportunities to evaluate candidate immunotherapies, such as adoptive T cell therapies (174, 175).

Transplantation of human liver tissue enables infection of the mouse host by human hepatotropic viruses. Because implantation of a liver fragment at an ectopic site does not fully recapitulate the architecture and function of the liver, protocols of orthotopic implantation have been developed. These methods follow the same principles as for the transplantation of human hematopoiesis (176): an immunodeficient strain to prevent immune rejection of the graft; engineering of the mouse to induce mouse liver injury and open the niche for human hepatocytes; and, in some instances, support from human growth factors (177). Several methods have been developed to eliminate mouse hepatocytes, relying on the expression of cytolytic proteins [e.g., overexpression of urokinase plasminogen activator under an albumin promoter in uPA mice (178, 179)] and/or inactivation of metabolic enzymes essential for hepatocyte homeostasis [e.g., inactivation of the fumarylacetoacetate hydrolase in *Fah*^−/−^ mice (180, 181)]. Upon injection of human hepatocytes, these mice support high levels of liver chimerism and are permissive for infection by the human hepatotropic viruses, HBV and HCV. To study human immune responses to these pathogens, mice can be dually transplanted with human hepatocytes and CD34^+^ HSPCs; human immune cells are recruited to the human liver in these chimeric mice and immune responses are triggered upon viral infections (177, 182, 183).

Finally, subcutaneous implantation of a fragment of human fetal lung contains all cell types naturally present in this tissue, and extends the permissiveness of the host mouse to respiratory pathogens with human tropism, including respiratory syncytial virus, Middle East respiratory syndrome coronavirus, and HCMV. This was recently accomplished by surgical implantation of a fragment of human lung tissue in BLT mice, resulting in the “BLT-L” model (184). Upon infection with HCMV, by direct injection in the lung implant, BLT-L mounted an antigen specific adaptive immune response, capable of controlling virus replication (184).

Thus, co-transplantation models greatly broaden the spectrum of human immune responses that can be studied in humanized mice. However, current approaches rely on highly specialized protocols and recipient mice, and should still be considered as prototypes under development.

## Conclusion

Tremendous progress has been accomplished since pioneering humanized mouse models were developed in the late 1980s. In the past decade, a flurry of new opportunities have been enabled by the optimization of recipient mouse strains and humanization protocols. The most advanced models support long-term multilineage human hematopoiesis, and recapitulate essential aspects of innate immunity and antigen-specific adaptive immunity. Furthermore, numerous hematologic diseases can now be modeled by xenotransplantation of primary patient-derived samples.

Despite this progress, limitations remain. Rigorous evaluation and comparison of the new models is needed, while supporting additional innovations that might drive transformative advances. Until one or more humanized mouse model fully recapitulates human immunity, we can enthusiastically but critically use the currently available strains to answer specific, clinically-relevant questions and hopefully inform the development of new, life-saving therapies (Box 2).

Box 2 **Which humanized mouse model is best for your studies?**

There is no one-size-fits-all model. Here are a few practical considerations:

1. Which mice and human cells/tissue do you have access to?
2. What question are you trying to answer?
3. Which cell types are important to answer your question?
  In addition to considering these practical questions, it is important to know what has been done before you. Take time to read the literature and critically analyze the experiments. If you are able to talk to the people who developed the model or to someone who has recently published on the model of interest, you may receive invaluable unpublished data and advice.

Finally, applying these criteria, we provide our own selection, subjective and probably slightly biased, of the models that we consider most suitable for specific applications.

**Hematopoiesis**

- HSPC biology: NSGW41, MISTRG, MISTRG6
- Erythropoiesis and thrombopoiesis: NSGW41, MISTRG, MISTRG6 (BM only, limited)
- Myelopoiesis: MISTRG, MISTRG6
- Hematologic malignancies: MISTRG, MISTRG6

**Innate immunity**

- Monocytes/macrophages: MISTRG, MISTRG6
- DCs: NSG (or any other strain, depending on additional requirements)
- Mast cells: NSG-SGM3
- Neutrophils: no suitable model available
- NK cells: SRG15, MISTRG
- Innate lymphoid cells: NSG (or any other strain, depending on additional requirements)

**Adaptive immunity**

BLT mice are generally considered as supporting more robust adaptive immunity than mice transplanted with HSPCs only. However, few direct comparisons have been reported and antigen-specific immune responses remain relatively weak or delayed, even in BLT mice. Importantly, the BLT protocol can be applied with any recipient mice.

Expression of HLA molecules by the mouse host qualitatively favors HLA-restricted immune responses. But the impact on the amplitude of responses remains to be rigorously quantified.

- B cells: BLT, SRG6, MISTRG6
- T cells: BLT, BRGST.

## Author Contributions

All authors listed have made a substantial, direct and intellectual contribution to the work, and approved it for publication.

## Conflict of Interest

The authors declare that the research was conducted in the absence of any commercial or financial relationships that could be construed as a potential conflict of interest.

## Abbreviations

AM: Alveolar macrophages
BLT: bone marrow, liver, thymus
BM: Bone marrow
BRG: BALB/c *Rag2*^−/−^ *Il2g*^−/−^
BRGF: BALB/c *Rag2*^−/−^ *Il2g*^−/−^ *Flt3*^−/−^
DCs: Dendritic cells
EBV: Epstein–Barr virus
EPO: Erythropoietin
G-CSF: Granulocyte colony-stimulating factor
GM-CSF: Granulocyte-macrophage colony-stimulating factor
GvHD: Graft-vs.-host disease
HCMV: Human Cytomegalovirus
HIV-1: human immunodeficiency virus 1
HLA: Human leukocyte antigen
HSPCs: Hematopoietic stem and progenitor cells
IL: Interleukin
MSCs: Mesenchymal stromal cells
MHC-I: Major histocompatibility complex class I
MHC-II: Major histocompatibility complex class II
MISTRG: M-CSF^h/h^ IL-3/GM-CSF^h/h^ SIRPα^h/m^ THPO^h/h^ RAG2^−/−^ IL2Rγ^−/−^
MISTRG6: M-CSF^h/h^ IL-3/GM-CSF^h/h^ SIRPα^h/m^ THPO^h/h^ RAG2^−/−^ IL2Rγ^−/−^ IL6^h/h^
NK: Natural killer
NOD: Non-obese diabetic
NSG: NOD/SCID/*Il2rg*^−/−^
NSGW41: NOD/SCID/*Il2rg*^−/−^ *Kit*^*W*41/*W*41^
PBMCs: peripheral blood mononuclear cells
PDX: Patient derived xenograft
RBCs: Red blood cells
SCF: Stem cell factor
SCID: severe-combined immunodeficient
TCR: T cell receptor
xGvHD: xenogeneic graft-vs.-host disease.
